# The influence of macro- and microelements in seminal plasma on diluted boar sperm quality

**DOI:** 10.1186/s13028-017-0279-y

**Published:** 2017-02-10

**Authors:** Maja Zakošek Pipan, Janko Mrkun, Breda Jakovac Strajn, Katarina Pavšič Vrtač, Janko Kos, Anja Pišlar, Petra Zrimšek

**Affiliations:** 1 Clinic for Reproduction and Large Animals, Veterinary Faculty, University of Ljubljana, Gerbičeva 60, 1000 Ljubljana, Slovenia; 2 Department for Environment and Animal Nutrition, Welfare and Hygiene, Veterinary Faculty, University of Ljubljana, Gerbičeva 60, 1000 Ljubljana, Slovenia; 3 Department of Pharmaceutical Biology, Faculty of Pharmacy, University of Ljubljana, Aškerčeva cesta 7, 1000 Ljubljana, Slovenia; 4 Department of Biotechnology, Jožef Štefan Institute, Jamova cesta 39, 1000 Ljubljana, Slovenia; 5 Institute for Preclinical Sciences, Veterinary Faculty, University of Ljubljana, Gerbičeva 60, 1000 Ljubljana, Slovenia

**Keywords:** Macroelements, Microelements, Boar semen, Liquid storage, Sperm quality

## Abstract

**Background:**

Growing evidence indicates that macro- and microelements in the seminal plasma of humans and various domestic animals are of great importance due to their roles in sperm metabolism, function, survival and oxidative stress. In the present study, we therefore determined the concentrations of macro- and microelements in fresh boar seminal plasma and their relation to sperm quality parameters after 3 days of liquid storage was assessed. Twenty ejaculates from eight boars were collected, and semen volume, concentration, sperm motility, morphology, tail membrane integrity, plasma membrane permeability, mitochondrial membrane potential and DNA fragmentation were determined on the day of collection (day 0) and day 3 (72 h) of storage at 15–17 °C. Seminal plasma was separated and the concentrations of macroelements (Na, K, Ca, and Mg) and microelements (Cu, Fe, Zn and Se) were determined.

**Results:**

After 3 days of storage Se levels correlated significantly with sperm motility, progressive motility and morphology, all of which are routinely used for semen evaluation. On day 3, Se levels also correlated with tail membrane integrity, viability and intact DNA (P < 0.05). The correlation coefficients showed that mitochondrial function was better preserved at higher levels of Zn, while higher levels of Cu decreased mitochondrial function, but led to the better preservation of DNA. It was also evident that higher levels of Fe were associated with higher proportions of live spermatozoa and of spermatozoa with normal morphology after 3 days of storage (P < 0.05), while higher levels of Ca and Mg in fresh seminal plasma were associated with lower percentages of progressive motile spermatozoa and with a decreased proportion of spermatozoa with intact DNA (P < 0.05). Multivariate analysis including microelements showed that Se significantly affected sperm quality parameters, mentioned above, after 3 days of storage.

**Conclusions:**

Macro- and microelements were associated with boar sperm quality and may be important biomarkers of boar sperm quality after liquid storage. Our results demonstrate that the evaluation of Se in fresh boar seminal plasma can serve as an additional tool in predicting sperm quality after storage.

## Background

The traditional semen-processing technology used in the swine industry is based on insemination of sows with chilled semen stored at 15–20 °C for 1–5 days after the addition of an appropriate extender [1]. However, the motility and viability of stored boar spermatozoa are diminished and alterations can occur in membrane permeability [2]. To maintain the high quality of semen for insemination, routine assessment based on the concentration and evaluation of motility and morphology is necessary [3]. However, their relation to in vivo fertility remains under discussion [4].

It is well-known that the ionic environment has a great influence on sperm function in humans [5]. The World Health Organization’s (WHO) guidance on the assessment of seminal plasma includes analysis of some macro- and microelements, such as Zn and Se, which are associated with sperm quality in humans due to their antioxidant properties [6, 7]. In men with Se deficiency, there is a loss of sperm motility, breakage at spermatozoa mid-piece level and increased incidence of spermatozoa head abnormalities [8]. In boars, the addition of Se to their diet resulted in contradictory results [9, 10]. In a recent study, Se supplementation did not affect sperm quantity or sperm quality [10], but increased levels of the predominant selenoprotein PHGPx, which is responsible for the integrity of mature spermatozoa [11]. Zn is crucial to the quality of spermatozoa and acts as a cofactor for many enzymes. Its deficiency results in disorders of testicular development and in spermatogenic failure [12, 13]. Macro- and microelements in boars have been studied [7, 14], but the nature of the influence of elements on semen characteristics needs further investigation.

The aim of the present study was to determine the concentrations of macro- and microelements in fresh boar seminal plasma and their association with sperm quality characteristics after 3 days of liquid storage.

## Methods

### Semen samples

Twenty ejaculates (2–3 per boar) obtained from eight mature and healthy boars of various breeds [two Slovenian Landrace line 11, one Slovenian landrace line 55, two Slovenian Large White, two Pietrain, and one Hibride line (54)] aged 12–24 months were included in the study. The boars included were of proven fertility with pregnancy rates of 69.5 ± 7.9% after insemination. Average litter size was 12.1 ± 0.3 piglets; 94.9% of born piglets were alive. The boars were housed in individual pens with straw bedding and received a standard balanced diet. Full ejaculates without the gel fraction were collected by the gloved-hand technique during routine farm operations at a local AI centre. Following collection, the filtered semen of each ejaculate was extended with Beltsville Thawing Solution (BTS, Truadeco, the Netherlands) at a ratio of 1:2.

### Sample preparation and basic semen characteristics

After arrival to the laboratory, an initial evaluation was performed of each diluted ejaculate, and those that fulfilled the requirements of >70% total motility, >35% progressive motility, <30% abnormal sperm morphology, and <20% proximal and distal cytoplasmic droplets were included in the study. Motility and progressive motility were determined using assisted semen analysis (Hamilton Thorne IVOS 10.2; Hamilton Thorne Research, MA, USA) with a Makler counting chamber (Sefi Medical Instruments, Haifa, Israel). Before analysis, semen samples were incubated in a water bath at 37 °C for 8 min. Thereafter, for each sample, 5 µl of diluted semen was mounted on a heated Makler counting chamber. Three randomly selected microscopic fields were scanned three times each, obtaining 9 scans for every semen sample. The mean of the three scans for each microscopic field was used for the statistical analysis. The software settings used in this study are summarized in Table 1.

**Table 1:** Software settings for Hamilton Thorne IVOS 10.2 used in this study

Hamilton Thorne settings	
Frames per second	60 Hz
Minimum cell size	10 pixels
Cell intensity	125
Straightness (STR) threshold	80%
Medium VAP cut-off	45 µm/s
Low VSL cut-off	15 µm/s
Low VAP cut-off	25 µm/s
Slow cells motile	No
Static head intensity	0.65–4.90
Static intensity gate	0.50–2.50
Magnification	1.89
Temperature of analysis	37 °C
Type of chamber	Makler

Concentrations were measured with a photometer (Photometer SDM 5, Minitüb, Germany). The morphology of 200 spermatozoa in diluted semen samples were assessed following eosin-nigrosin staining (Morphology stain, Society for Theriogenology).

Semen analysis was performed on the day of collection (day 0) and on day 3 (72 h) of semen preservation. Semen samples were stored on day 0 for 3 days in closed plastic containers in a thermal box at 15–17 °C with constant gentle agitation.

### Additional semen analyses

#### Hypoosmotic swelling test (HOST)

A HOST was applied to evaluate tail membrane integrity as a test of sperm function. First, 10 μl of the semen sample was mixed gently with 90 μl of hypoosmotic solution (150 mOsm/kg sodium citrate × 2H_2_O and fructose) at 37 °C. After 1 h of incubation at 37 °C in a warm water tub, 200 spermatozoa per sample were examined under a light microscope at a magnification of 400×. Spermatozoa were considered HOST positive if they showed signs of swelling, as described by Hishinuma and Sekine [15].

#### Assessment of membrane modifications using the Yo-Pro-1/PI assay

Staining spermatozoa with Yo-Pro-1/PI was used to detect changes in plasma membrane permeability as described by Idziorek et al. [16]. After staining, different cell populations were distinguished using a flow cytometer (FACSCalibur, BD Bioscience, San Jose, CA, USA). Channel FL-1 was used to detect green fluorescence (Yo-Pro-1), while channel FL3 was used to detect red fluorescence (PI).

Sheath flow rate was set at 6–24 µl/min, and a minimum of 20,000 events were recorded. The analyser threshold was adjusted on the electronic volume channel to exclude subcellular debris and cell aggregates. Light signals were converted into electrical signals by a photo detector and evaluated by a software programme (Flow Jo, Ashland, USA).

The cell population was separated into three groups: live cells, showing no fluorescence (Yo-Pro-1 −/PI −); (early) apoptotic cells, showing an incrementally higher level of green fluorescence (Yo-Pro-1 +/PI −); and late apoptotic/dead (Yo-Pro-1 +/PI +) and necrotic cells (Yo-Pro-1 −/PI +), showing red fluorescence.

#### Assessment of mitochondrial membrane potential

Mitochondrial membrane potential (MMP) is an indicator of sperm functionality. MitoTracker Red is a fluorescent probe suitable to differentiate spermatozoa with deteriorated mitochondria (in apoptotic cells) from that of living spermatozoa [17]. A slightly modified protocol was used for this study. Briefly, ten million sperm cells washed in PBS (centrifuged at 400*g* for 30 min) were added to 1 ml of PBS. Next, 10 μl of stain MitoTracker Red (500 nM) (Invitrogen) was added, and the tubes were gently mixed and incubated for 30 min in the dark at 37 °C. The fluorescence signal was monitored at the FL2 channel of a flow cytometer (FACSCalibur, BD Bioscience, San Jose, CA, USA). Sheath flow rate was set at 6–24 µl/min, and a minimum of 20,000 events were recorded. The analyser threshold was adjusted on the electronic volume channel to exclude subcellular debris and cell aggregates. Spermatozoa incubated in 50 μM carbonyl cyanide *m*-chlorophenyl hydrazone (CCCP), known to reduce mitochondrial membrane potential, were used as a control.

MitoTracker dye positive sperm events indicated cells with active membrane-polarized mitochondria, whereas cells with reduced MitoTracker fluorescence were those whose mitochondria had reduced mitochondrial transmembrane potential.

#### DNA fragmentation

DNA integrity was evaluated using a commercial test (Sperm Sus-Halomax; Halotech DNA SL, Spain) based on the Sperm Dispersion test and specifically designed for boar spermatozoa. The semen samples were processed according to the manufacturer’s instructions and stained with a commercial fluorescence microscopy green staining kit (Halotech DNA, Spain) according to the instructions. The fluorescence stained sample was placed into the well of a slide prior to microscopic assessment. Sperm chromatin dispersion was evaluated using a fluorescence filter (Olympus U-MNIBA3; excitation at 497 nm and emission at 520 nm) with 400× magnification (Olympus BX40). A minimum of 300 spermatozoa were counted per semen sample.

The spermatozoa were classified into three categories according to the shape of the halo effect: (1) normal halo, a clearly visible halo around the head similar to the diameter of the core; (2) a small or absent halo, a small halo spotted around the head or a complete absence of a halo; and (3) a large scattered halo, a very large and scattered halo around the head. Based on the halo effect, spermatozoa were then classified into two categories: spermatozoa with a normal halo effect, denoting intact DNA and spermatozoa with a small, absent or large scattered halo effect, denoting impaired DNA.

#### Analysis of macro- and microelements

A semen sample was centrifuged at 800*g* for 10 min at room temperature. The supernatant was removed and centrifuged again at 13,000*g* for 15 min at 4 °C to separate seminal plasma, which was then aliquoted and frozen at −80 °C until assayed for Na, Mg, Ca, K, Fe, Cu, Zn and Se. Analysis of the elements was performed as described previously [18] with a slight modification. Microwave digestion of the samples was performed using an MARS 5 Microwave Acceleration Reaction System (CEM, Matthews, NC). A total 2 ml of a sample was transferred into a 100 ml Teflon vessel and 3 ml 65% nitric acid, 0.5 ml 30% hydrogen peroxide and 4.5 ml Milli-Q water were added. The samples were digested in a closed 12 vessel microwave system at 200 °C for 30 min. After cooling to room temperature, the solutions were diluted with Milli-Q water and the concentrations of elements were determined by inductively coupled plasma mass spectrometry (Varian 820-MS, Mulgrave, Australia). Argon was used as the carrier gas and the isotopes ^23^Na, ^24^Mg, ^44^Ca, ^39^K, ^57^Fe, ^63^Cu, ^66^Zn, ^78^Se were selected as analytical masses in the ICP-MS normal sensitivity mode. A Collision Reaction Interface (CRI) was used for the measurements of Se to reduce common polyatomic interferences.

### Statistical analysis

Data are presented as the mean ± standard deviation (SD) and median. The normal distributions of data were tested by the Shapiro–Wilk test. In cases of normal distribution, parametric tests were performed; in case of non-normal distributed data, non-parametric tests were performed. Spearman or Pearson rank correlation coefficients were used to determine the correlation between sperm quality characteristics on days 0 and 3 and to evaluate the association between the concentration of trace elements on day 0 and the sperm quality characteristics on day 3. Statistical analyses were performed using SPSS (IBM SPSS Statistics 22). P < 0.05 was considered significant. A multivariate linear regression model was used to assess the association between microelements (Fe, Cu, Zn and Se) and each sperm quality characteristic on day 3 of semen storage. Analyses were performed in R Statistical Software (version 3.1.1).

## Results

### Characteristics of sperm quality

The mean concentration of spermatozoa in semen ejaculates was 319.5 ± 116.1 × 10^6^ ml. Measurement characteristics of sperm quality were made for fresh semen samples (day 0) and for semen samples after 3 days of storage (day 3) (Table 2). The values of sperm quality characteristics differed significantly between days 0 and 3 for all sperm quality characteristics (P < 0.05), except for proximal and distal droplets (P > 0.05) (Table 2).

**Table 2:** Values (mean ± SD, median) of boar sperm quality characteristics on day 0 and after 3 days of liquid storage (n = 20)

Semen parameters (%)	Day 0		Day 3	
	Mean ± SD	Median	Mean ± SD	Median
Motility	80.5 ± 4.4	80.1	60.4 ± 11.9	62.6
Progressive motility	47.2 ± 7.7	47.3	30.0 ± 7.8	29.6
Normal sperm morphology	75.6 ± 6.9	76.4	59.8 ± 9.6	57.2
Proximal droplets	4.2 ± 3.5	3.9	4.4 ± 3.4	4.7
Distal droplets	5.5 ± 3.0	4.6	5.6 ± 2.8	4.9
Acrosome reacted spermatozoa	12.3 ± 4.5	11.5	27.2 ± 7.7	25.9
Capacitated spermatozoa	4.1 ± 2.7	3.5	15.3 ± 4.7	15.0
Impaired tail membrane integrity	14.4 ± 3.8	14.7	36.6 ± 9.8	34.9
Membrane potential				
Live spermatozoa	67.3 ± 7.0	66.9	39.1 ± 9.4	40.2
Dead spermatozoa	25.1 ± 6.7	25.6	47.1 ± 9.9	44.9
Apoptotic spermatozoa	7.6 ± 4.6	6.8	13.8 ± 3.5	13.5
Decreased mitochondrial potential	4.8 ± 7.6	3.4	26.1 ± 25.9	21.1
Intact DNA	90.8 ± 6.6	93.3	82.9 ± 6.9	83.8

### Values of elements measured in seminal plasma on day 0

Values of elements measured in seminal plasma on day 0 are shown in Table 3.

**Table 3:** Concentration of elements (mean, median, minimum and maximum) in boar seminal plasma (n = 20)

	Mean	Median	Minimum	Maximum
Na (mmol/l)	96.3	96.4	81.3	106.8
Mg (mmol/l)	2.10	1.78	0.62	4.98
K (mmol/l)	13.2	12.9	11.7	14.8
Ca (µmol/l)	179.4	161.3	76.8	285.9
Fe (µmol/l)	2.71	2.66	1.73	4.58
Cu (µmol/l)	0.94	0.97	0.49	1.35
Zn (µmol/l)	212.4	194.1	59.4	475.7
Se (nmol/l)	78.6	71.5	55.7	150.2

### Correlation between sperm quality characteristics on days 0 and 3

Correlation coefficients were determined for sperm characteristics on days 0 and 3 of liquid storage (Table 4).

**Table 4:** Correlations (r value) between sperm quality characteristics on day 0 and sperm quality characteristics after 3 days of storage (n = 20)

Day 0	Day 3							
	Motility	Progressive motility	Normal sperm morphology	Acrosome reacted spermatozoa	Dead spermatozoa	Apoptotic spermatozoa	Intact DNA	Impaired tail membrane integrity
Motility	0.504*	0.466*	0.036	−0.415	−0.357	0.136	0.327	−0.469*
Progressive motility	0.498*	0.384	0.140	−0.147	−0.458*	−0.560*	0.554*	−0.372
Normal sperm morphology	0.325	0.367	0.794*	−0.444*	−0.234	−0.245	0.111	−0.476*
Proximal droplets	−0.212	−0.337	−0.465	−0.166	0.000	0.165	−0.038	0.270
Distal droplets	−0.193	−0.084	−0.153	0.090	0.311	0.099	0.040	0.063
Capacitated spermatozoa	0.138	0.243	0.494*	−0.471*	−0.501*	0.421	0.613*	−0.573*
Impaired tail membrane integrity	−0.027	−0.129	−0.380	0.371	−0.093	0.183	−0.485*	0.242
Live spermatozoa	0.582*	0.502*	−0.011	−0.119	−0.007	−0.322	−0.274	0.150
Apoptotic spermatozoa	−0.392	−0.474*	−0.304	0.072	−0.145	0.494*	−0.652*	−0.284
Decreased mitochondrial potential	−0.027	−0.129	−0.380	0.371	−0.093	0.183	−0.485*	−0.027
Intact DNA	0.342	0.500*	0.199	−0.347	−0.340	−0.571*	0.946*	−0.618*

Only sperm quality characteristics that showed at least one correlation are included in the table

*P values <0.05

Intact DNA on day 3 correlated with the highest number of sperm quality characteristics in fresh semen. Significant positive correlations were observed with progressive motility and capacitation (P < 0.05), whereas correlations with impaired tail membrane integrity, apoptotic spermatozoa and decreased mitochondrial membrane potential on day 3 were negative (P < 0.05). High positive correlation was also observed for intact DNA between day 0 and day 3. Progressive motility on day 3 correlated with four sperm quality characteristics on day 0. Significant positive correlations were observed with motility, the proportion of live spermatozoa and intact DNA (P < 0.05), whereas correlation with the proportion of apoptotic spermatozoa was negative (P < 0.05). Impaired tail membrane integrity also correlated with motility and intact DNA, but correlations were negative (P < 0.05). There was also a negative correlation between impaired tail membrane integrity on day 3 and the proportion of morphologically normal and capacitated spermatozoa on day 0 (P < 0.05). Sperm motility and the proportion of apoptotic spermatozoa on day 3 correlated with three sperm quality characteristics on day 0. Sperm motility on day 3 correlated positively with sperm motility, progressive motility and proportion of live spermatozoa on day 0 (P < 0.05). The proportion of apoptotic spermatozoa on day 3 correlated positively with that of apoptotic spermatozoa on day 0 (P < 0.05), whereas correlations with intact DNA and progressive motility on day 0 were negative (P < 0.05). On day 3, normal sperm morphology correlated positively and acrosome reacted spermatozoa negatively with normal sperm morphology and capacitated spermatozoa on day 0 (P < 0.05). Late apoptotic/dead spermatozoa on day 3 correlated negatively with progressive motility and the proportion of capacitated spermatozoa (P < 0.05).

Capacitated spermatozoa, live spermatozoa and mitochondrial membrane potential on day 3 did not correlate with any sperm quality characteristics on day 0 (P > 0.05).

### Correlations between element concentrations in seminal plasma on day 0 and sperm quality characteristics on day 3

Correlations between element concentrations in seminal plasma on day 0 and sperm quality characteristics on day 3 are summarized in Table 5; only correlations with elements that showed at least one significant correlation are presented.

**Table 5:** Correlations (r value) between the concentrations of elements in boar seminal plasma measured on day 0 and sperm quality characteristics on day 3 (n = 20)

Semen parameters on day 3	Element concentration in seminal plasma on day 0					
	Mg (mmol/l)	Ca (µmol/l)	Fe (µmol/l)	Cu (µmol/l)	Zn (µmol/l)	Se (nmol/l)
Motility	−0.168	−0.325	0.035	0.042	−0.350	0.674*
Progressive motility	−0.497*	−0.436*	0.177	0.140	−0.087	0.563*
Normal sperm morphology	0.136	0.360	0.555*	−0.126	0.430	0.757*
Proximal droplets	−0.190	−0.231	−0.090	−0.106	0.113	−0.252
Distal droplets	−0.276	−0.157	−0.133	0.225	0.174	−0.045
Acrosome reacted spermatozoa	0.000	0.242	0.208	0.132	−0.007	−0.406*
Capacitated spermatozoa	0.236	−0.144	−0.144	−0.254	−0.117	0.138
Impaired tail membrane integrity	0.147	0.463*	0.119	−0.215	0.187	−0.716*
Live spermatozoa	0.091	0.237	0.496*	−0.323	−0.266	0.467*
Dead spermatozoa	0.022	−0.146	−0.547*	0.207	0.200	−0.455*
Decreased mitochondrial potential	−0.174	−0.438	−0.414	0.582*	−0.625*	−0.303
Intact DNA	−0.475*	−0.567*	0.149	0.506*	0.240	0.473*

*P values <0.05

Selenium in the seminal plasma of fresh boar semen correlated with many sperm quality characteristics after 3 days of storage. Significant positive correlations of Se were observed with motility, progressive motility, normal sperm morphology, live spermatozoa and spermatozoa with intact DNA while negative correlations were found for acrosome reacted spermatozoa, dead spermatozoa and spermatozoa with impaired tail membrane integrity on day 3 (all P values <0.05) (Table 5). Iron showed a positive correlation with normal sperm morphology and the proportion of live spermatozoa and a significant negative correlation with the proportion of dead spermatozoa (all P values <0.05), as observed for Se. Ca correlated with three characteristics as for Se, but the correlations were inverse; a positive correlation was observed with impaired tail membrane integrity and a negative correlation with the proportion of progressive motility and DNA fragmentation (all P values <0.05). Magnesium showed the same correlation as did Ca, except for lack of correlation between Mg and impaired tail membrane integrity (P > 0.05).

Copper showed a significant positive correlation while Zn showed a significant negative correlation with decreased mitochondrial membrane potential (P < 0.05). Copper also showed a significant positive correlation with intact DNA (P < 0.05). There was no association of either K or Na with sperm quality characteristics (P > 0.05).

Taken together, DNA fragmentation correlated with the highest number of elements; it negatively correlated with Mg (P < 0.05) and Ca (P < 0.05) and positively correlated with Cu (P < 0.05) and Se (P < 0.05).

### Results of multivariate regression model including microelements

Association between microelements in fresh seminal plasma and sperm quality parameters after 3 days of storage was analysed by multivariate linear regression model. Selenium was significantly associated with sperm motility (P = 0.012), progressive motility (P = 0.018), morphology (P = 0.004) and acrosomal reaction of the spermatozoa (P = 0.007). Tail membrane integrity was also affected by Se (P = 0.003) as well as by Fe (P = 0.033), while the proportion of live spermatozoa after storage was significantly associated with Cu (P = 0.032). Moreover, the proportion of live spermatozoa and spermatozoa with intact DNA tended to be higher in semen with higher concentration of Se in fresh seminal plasma (P = 0.056 and P = 0.084, respectively).

## Discussion

The results of the present study show correlations between macro- and microelements in fresh boar seminal plasma and sperm quality characteristics after 3 days of liquid storage. Microelement concentrations, especially for Se, were associated with sperm quality characteristics.

The concentrations of macro- and microelements were similar to those in previous studies conducted on fresh boar semen [7, 14]. It has been shown that K helps to preserve sperm motility and is added to several commercial extenders [1] and that Na correlates with normal sperm morphology [14]. In our study, semen was extended with BTS for the purposes of short term storage. BTS contains Na-citrate, Na-bicarbonate, KCl, EDTA, glucose and antibiotics; therefore, no correlations were found between sperm quality characteristics and the concentrations of Na and K. The concentrations of Na and K used were higher in our study than in the study conducted by Lopez Rodriguez et al. [14] because they used fresh boar semen without extenders.

In the male reproductive system, Fe can display either positive or negative roles, depending on its concentration. An increased concentration of Fe in the testes was associated with oxidative damage of lipids, proteins, and DNA [19]. However, Fe deficiency reduced the activity of Fe-containing and Fe-depending enzymes [7]. Massanyi et al. [7] found a strong positive correlation between Fe and Zn in fresh boar semen and it was recently demonstrated in bulls that adding FeCl_2_ to diluted semen samples at concentrations above 50 μmol/l leads to a significant decrease of sperm motility and mitochondrial activity. However, concentrations below 10 μmol/l FeCl_2_ stimulated spermatozoa activity, as shown by a significant preservation of motility and viability characteristics in diluted bull spermatozoa [20]. In our study, where the mean Fe concentration in boar seminal plasma was 2.71 μmol/l (minimum, 1.73 μmol/l; maximum, 4.58 μmol/l), higher Fe levels were correlated with a higher level of normal sperm morphology and live spermatozoa after storage. From the multivariate analysis it was found that Fe affected also tail membrane integrity. Because spermatozoa are under increased oxidative stress during storage [2], low levels of Fe, which lead to lower activity of Fe-dependent enzymes such as catalase [7], could lead to increased lipid peroxidation in boar spermatozoa and result in reduced viability.

A significant negative correlation was observed between Zn concentrations on day 0 and decreased mitochondrial membrane potential on day 3, indicating that higher levels of Zn better preserved mitochondrial function. However, the higher levels of Cu decreased mitochondrial function, but better preserved DNA. Copper is essential for many enzymes such as superoxide dismutase, which is involved in protecting cells against oxygen free radicals [21]. This may be the reason for the higher percentage of spermatozoa with intact DNA in our study. Copper is also needed for cytochrome c oxidase, which is responsible for energy supply and for cellular and humoral immunity [21]. However, elevated Cu concentrations reduce glycolysis, which may be the reason for the decreased mitochondrial potential that manifested in the decrease of sperm motility [22]. In our study, among microelements, Cu was found to be significantly associated with the proportion of live spermatozoa. After the addition of Zn to human semen samples, a significantly higher percentage of sperm with intact DNA and normal mitochondrial function was found [23]. We also noted a trend of positive association for Zn with normal sperm morphology after storage, but the correlation was not significant (P = 0.058). A similar correlation was found for fresh human semen [24]. A previous study in boars did not find any correlation between Zn in fresh seminal plasma and sperm quality characteristics, but there was a negative correlation with abnormal tails [14]. It has been suggested that Zn affects sperm quality in different ways, making it difficult to associate it with a single parameter [14]. Zinc is able to protect spermatozoa against oxidative stress [24] and is seen as a better preserver of mitochondrial function since mitochondria are the major site of intracellular formation of reactive oxygen species [25].

Selenium was correlated with most of the sperm quality characteristics. The higher preservation of membrane integrity and normal sperm morphology observed in our study are in agreement with a study conducted on fresh boar semen by Lopez Rodriguez et al. [14], where higher levels of Se were associated with less membrane damage and fewer proximal droplets. Selenium participates in a variety of physiological functions as an integral part of a range of selenoproteins. It is also an important component of the enzyme glutathione peroxidase that protects cell membranes against the adverse effects of lipid peroxides [26], thereby preserving the structural integrity of the spermatozoa plasma membrane [27]. Our study also confirmed these results with correlations between Se and the preservation of membrane integrity, which is also reflected in preservation or increase of ATP in spermatozoa, leading to improved sperm motility and progressive motility [28]. This is again confirmed in our study by the positive correlations between Se and sperm motility and progressive motility. On the basis of multivariate analysis the association between Se in fresh seminal plasma and sperm quality characteristic, mentioned above, was confirmed. Low levels of Se can lead to higher levels of active peroxides during the final stages of spermatogenesis, which can cause oxidative injuries that could accumulate and lead to delayed impairment of viability [29]. Impaired spermatogenesis arising from Se deficiency has been reported in several animal species including boars [28], where changes in mitochondria, decreased sperm ATP concentration and increased percentage of immature spermatozoa were noted in boars fed a low-selenium diet [28]. It was suggested that Se plays a role in establishing the number of spermatozoa reserves and Sertoli cells. Boars fed a low Se diet also possessed spermatozoa with lower motility and increased abnormal morphology [28]. Although in the present study, Se intake was the same in all boars, the content of Se in seminal plasma varied. The same was shown in study conducted on fresh boar semen by Lasota et al. [30]. Even though in our study, semen samples that had a higher content of Se had better preserved sperm quality following 3 days of storage, it was found that when sodium selenite was added directly to the boar extender, sperm motility was reduced [28]. This could be because although Se and Zn can improve sperm quality, they may be harmful above certain levels [31].

Calcium and Mg levels correlated negatively with sperm progressive motility and the increased proportion of spermatozoa with intact DNA. Kasperczyk et al. [32] found a positive correlation between the levels of interleukin-12 and Mg, suggesting that Mg may indirectly promote the development of oxidative stress, thereby leading to impaired DNA and decreased sperm motility. Calcium acts as the trigger of the acrosome reaction in mammalian spermatozoa, and there is substantial evidence that it is differentially involved in sperm motility depending on the stage of sperm maturation, thereby causing decreased sperm motility [33].

The higher levels of Se were consistent with better conserved sperm quality characteristics after 3 days of storage. Infertile men have lower levels of Se in their seminal plasma [34]. Since traditional estimates of sperm quality are not sufficiently sensitive to differentiate between samples that differ in terms of predicting quality following storage [35], measurements of Se in boar seminal plasma could be of value in predicting sperm quality characteristics of semen following storage. Recently, new markers of sperm function that could enable better prediction of fertilizing ability in boars have been sought; the tumour necrosis factor, TNF-α [36], and superoxide dismutase [37], each measured in diluted fresh seminal plasma, were found to be valuable predictors of sperm quality after 3 days of storage. Our study shows that the concentration of Se correlated not only with sperm motility, progressive motility and morphology, which are all used routinely for semen evaluation but also with tail membrane integrity, sperm viability and intact DNA. These methods are also very effective in evaluating semen and can provide additional information about sperm fertility. The results of our study indicate that measuring Se in fresh seminal plasma can be helpful in predicting sperm quality following 3 days of storage.

Although higher concentrations of Se in fresh seminal plasma correlated with improved sperm quality after 3 days of storage in our study, current evidence suggests that optimal levels of Se is needed for good sperm quality [38].

Very high levels of Se could cause an opposite effect and reduce sperm quality. To predict sperm quality after 3 days of storage with Se, reference values should be established on larger numbers of semen samples. Se in fresh seminal plasma must be evaluated as possible predictors of sperm quality after storage. Based on the sperm quality after storage, the highly predictive values of Se could be used in the future as an additional tool in semen evaluation.

## Conclusions

An analysis of macro- and microelements in fresh boar seminal plasma demonstrated an association with boar sperm quality and therefore provides additional information about sperm quality after liquid storage. Due to the significant correlations between Se concentrations in fresh seminal plasma and semen parameters after storage, the evaluation of Se in fresh boar seminal plasma could serve as an additional tool in predicting sperm quality after storage.
